# Impaired differentiation of macrophage lineage cells attenuates bone remodeling and inflammatory angiogenesis in *Ndrg1* deficient mice

**DOI:** 10.1038/srep19470

**Published:** 2016-01-18

**Authors:** Kosuke Watari, Tomohiro Shibata, Hiroshi Nabeshima, Ai Shinoda, Yuichi Fukunaga, Akihiko Kawahara, Kazuyuki Karasuyama, Jun-ichi Fukushi, Yukihide Iwamoto, Michihiko Kuwano, Mayumi Ono

**Affiliations:** 1 Department of Pharmaceutical Oncology, Graduate School of Pharmaceutical Sciences, Kyushu University, Fukuoka 812-8582, Japan; 2 Department of Diagnostic Pathology, Kurume University Hospital, Kurume 830-0011, Japan; 3 Department of Orthopedic Surgery, Graduate School of Medical Sciences, Kyushu University, Fukuoka 812-8582, Japan; 4 Cancer Translational Research Center, St. Mary’s Institute of Health Sciences, Kurume 830-8543, Japan

## Abstract

*N-myc downstream regulated gene 1 (NDRG1)* is a responsible gene for a hereditary motor and sensory neuropathy-Lom (Charcot–Marie–Tooth disease type 4D). This is the first study aiming to assess the contribution of *NDRG1* to differentiation of macrophage lineage cells, which has important implications for bone remodeling and inflammatory angiogenesis. *Ndrg1* knockout (KO) mice exhibited abnormal curvature of the spine, high trabecular bone mass, and reduced number of osteoclasts. We observed that serum levels of macrophage colony-stimulating factor (M-CSF) and macrophage-related cytokines were markedly decreased in KO mice. Differentiation of bone marrow (BM) cells into osteoclasts, M1/M2-type macrophages and dendritic cells was all impaired. Furthermore, KO mice also showed reduced tumor growth and angiogenesis by cancer cells, accompanied by decreased infiltration of tumor-associated macrophages. The transfer of BM-derived macrophages from KO mice into BM-eradicated wild type (WT) mice induced much less tumor angiogenesis than observed in WT mice. Angiogenesis in corneas in response to inflammatory stimuli was also suppressed with decreased infiltration of macrophages. Taken together, these results indicate that NDRG1 deficiency attenuates the differentiation of macrophage lineage cells, suppressing bone remodeling and inflammatory angiogenesis. This study strongly suggests the crucial role of NDRG1 in differentiation process for macrophages.

The N-myc downstream regulated gene (NDRG) family of proteins consists of 4 members, NDRG1–4, which are evolutionarily well-conserved. Among the NDRG proteins, NDRG1 was first discovered because its expression is repressed by the proto-oncogenes *MYCN* and *MYC*. NDRG1 was designated as a reducing agent and tunicamycin-responsive protein (RTP), human differentiation-related gene 1 (DRG1), and reduced in tumor p42 (rit 42). NDRG1 is also called Cap43, and its expression is specifically induced by calcium or nickel^1^. NDRG1 is involved in several normal biological processes, including embryogenesis, development, cell growth and differentiation, migration, stress responses and immunity^2^, and many important studies of NDRG1 function have been published since its discovery. In particular, NDRG1 is intimately involved in multiple stages of differentiation, including placentation^3^ and trophoblast formation^4^, as well as in the morphogenesis of various organs^5^,^6^,^7^. However, the precise molecular and cellular functions of NDRG1 have not been fully defined.

NDRG1 has been implicated in lipid synthesis and myelination^8^. *Ndrg1* knockout (KO) mice retain complex motor skills but exhibit muscle weakness, the progressive demyelination of nerves^9^ and Schwann cell dysfunction^10^. However, NDRG1 participates in important immune system functions, including anaphylaxis, defense against bacterial pathogens, inflammation, and wound healing^11^. The expression of NDRG1 allows bone marrow (BM)-derived mast cells to transform into their mature counterparts^12^. *Ndrg1* KO mice exhibit a decreased number of mast cells that display impaired degranulation, indicating an attenuated immune response to antigens^13^. Together, these data indicate that NDRG1 may modulate various differentiation processes in the nervous and immune systems.

NDRG1 is known as a metastasis and oncogenic suppressor in cancers of the brain, breast, colon, esophagus, pancreas and prostate, and also as an oncogenic promoter in cancers of the kidney, liver, mouth, skin and stomach^2^,^11^, suggesting that the effects of NDRG1 as a tumor suppressor or tumor promoter depends on tumor type. Consistent with these findings, we have previously reported that the overexpression of NDRG1 in pancreatic cancer cells suppresses tumor growth and angiogenesis^14^,^15^, while NDRG1 overexpression in stomach cancer cells promotes tumor growth and angiogenesis^16^,^17^.

BM, which supplies various progenitor cells, is also an important tissue for the growth and survival of cancer cells^18^. These progenitor cells can be recruited to the primary tumor site, where they differentiate and become part of the tumor stroma. In particular, macrophages are known to play crucial roles in the growth, angiogenesis and metastasis of cancer cells^19^,^20^. However, it remains unclear whether NDRG1 can modulate tumor progression by acting on progenitor cells, including macrophages.

In the present study, we asked whether *NDRG1* deficiency in a host could critically affect biological and pathological processes, including development, differentiation and tumorigenesis, and we investigated whether and how *NDRG1* deficiency could modulate bone remodeling and inflammatory angiogenesis. The possible role of NDRG1 in these processes was discussed in the context of the differentiation and activation of macrophage lineage cells.

## Results

### *Ndrg1* KO mice shows decreased serum levels of M-CSF and macrophage-producing cytokines

**Figure 1:**
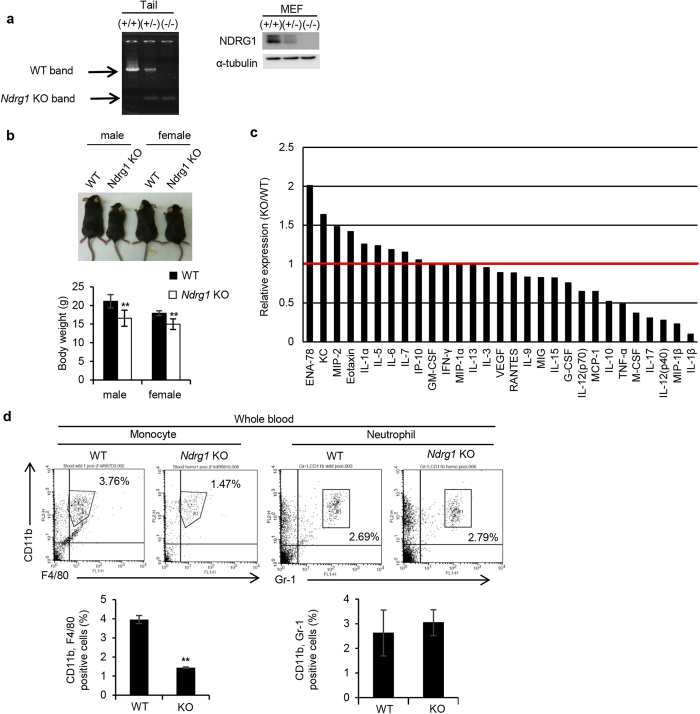
*Ndrg1* knockout mice shows decreased serum levels of M-CSF, macrophage-producing cytokines and low macrophage population in serum. (**a**) Establishment of *Ndrg1* KO (−/−) mice. PCR analysis of genomic DNA isolated from tails of each group of mice (left). Western blot analysis of cell lysates prepared from MEFs derived from each group of mice (right). Full-length blot are presented in Supplementary Figure 1. (**b**) Comparison of body size (top) and weight (bottom) between male and female WT and *Ndrg1* KO mice at 5 weeks of age (*n* = 5 per genotype). (**c**) Multiplex suspension array analysis of serum levels of various growth factors, cytokines and chemokines. Relative expression levels are presented. The data were normalized by the WT expression levels of each factor. (**d**) Flow cytometric analysis of monocyte and neutrophil populations in whole blood. The percentages of F4/80+, CD11b+ cells (monocytes) and Gr-1+, CD11b+ cells (neutrophils) in the blood are shown (*n* = 3 per genotype). Each bar is an average ± SD, **P < 0.01 versus WT mice (two-tailed Student’s t-test).

*Ndrg1* KO (−/−) mice were established by gene targeting, and these mice exhibited progressive demyelination of peripheral nerves^9^. Both tail and embryonic fibroblasts (MEF) showed an almost complete loss of NDRG1 gene and protein expression in *Ndrg1* KO mice (Fig. 1a). Both male and female *Ndrg1* KO mice were smaller in total body size and weight than WT mice (Fig. 1b).

Then, we compared serum levels of various growth factors, cytokines and chemokines between *Ndrg1* KO and WT mice by a Multiplex suspension array. Figure 1c shows that serum levels of cytokines and chemokines, including IL-10, tumor necrosis factor (TNF)-α, IL-12 (p40), the macrophage inflammatory proteins (MIP)-1β and IL-1β, which are mainly produced by macrophages, were decreased to approximately 50% or less in *Ndrg1* KO mice compared to WT mice. Furthermore, serum levels of macrophage colony-stimulating factor (M-CSF), also known as CSF1, were also much lower in *Ndrg1* KO mice (Fig. 1c).

We next compared the number of monocytes in whole blood samples between *Ndrg1* KO and WT mice. CD11b+, F4/80+ monocytes comprised 3.76% and 1.47% of the whole blood cell population in WT and *Ndrg1* KO mice, respectively (Fig. 1d). The number of CD11b+, F4/80+ cells in the blood was significantly decreased by *Ndrg1* knockdown. However, CD11b+, Gr-1+ neutrophil population was similar between WT and *Ndrg1* KO mice (Fig. 1d).

### NDRG1 deficiency retards the differentiation of bone marrow cells into macrophages and dendritic cells

**Figure 2:**
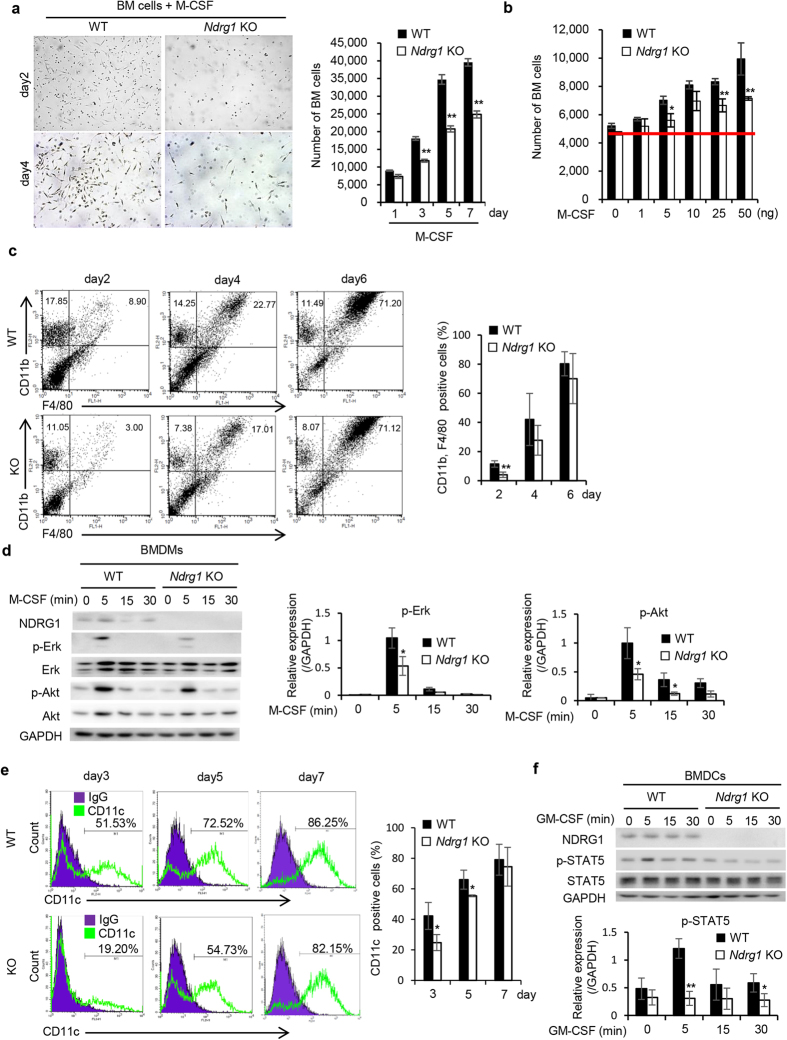
Reduced proliferation and differentiation of bone marrow cells from *Ndrg1* KO mice. (**a**) Morphological observations of BM cells at days 2 and 4 after stimulation of M-CSF (left) (original magnification ×40). Comparison of cell proliferation rates between BM cells from WT and *Ndrg1* KO mice in the presence of M-CSF (20 ng/mL) (right) (*n* = 3 per genotype). (**b**) Dose-response curves of cell proliferation in BM cells in the presence of the indicated concentrations of M-CSF (*n* = 3 per genotype). (**c**) Flow cytometric analysis of CD11b+, F4/80+ macrophage population in BM cells at days 2, 4 and 6 in the presence of M-CSF (20 ng/mL) (left). The percentages of CD11b+, F4/80+ cells (macrophages) are shown (right) (*n* = 3 per genotype). (**d**) Western blots show time kinetics for the phosphorylation of Erk and Akt in BMDMs at day 2 when cultured in serum free medium for 6 h, and then stimulated with 30 ng/mL M-CSF for indicated time. The right panel shows the quantification of expression levels of p-Erk and p-Akt normalized to the expression levels of GAPDH (*n* = 3 per genotype). Full-length blot are presented in Supplementary Figure 2. (**e**) Flow cytometric analysis of CD11c+ dendritic cell population in BM cells at days 3, 5 and 7 in the presence of GM-CSF (25 ng/mL) (left). The percentages of CD11c + cells (dendritic cells) are shown (right) (*n* = 3 per genotype). (**f**) Western blots show time kinetics for the phosphorylation of STAT5 in BMDCs at day 2 when cultured in serum free medium for 6 h, and then stimulated with 10 ng/mL GM-CSF for indicated time. The lower panel shows the quantification of expression levels of p-STAT5 normalized to the expression levels of GAPDH (*n* = 3 per genotype). Full-length blot are presented in Supplementary Figure 3. Each bar is an average ± SD, *P < 0.05; **P < 0.01 versus WT mice (two-tailed Student’s t-test).

M-CSF is known to be the primary growth factor that regulates the survival, proliferation and differentiation of macrophages and other mononuclear phagocytotic lineage cells^21^. We next examined whether *NDRG1* deficiency in BM cells affected cell proliferation and differentiation stimulated with M-CSF. BM cells from *Ndrg1* KO mice showed significantly reduced M-CSF-induced cell proliferation rates than those from WT mice (Fig. 2a). As shown in Fig. 2b, M-CSF dose-dependently promoted the proliferation of BM cells in both WT and *Ndrg1* KO mice. However, the proliferation rates of BM cells from *Ndrg1* KO mice were significantly lower than those of BM cells from WT mice.

M-CSF could induce differentiation of BM cells from both WT and *Ndrg1* KO mice into CD11b+, F4/80+ macrophages at day 6, 71.20% and 71.12% respectively (Fig. 2c). However, the numbers of CD11b+, F4/80+ macrophages at days 2 was significantly reduced in BM cells from *Ndrg1* KO mice than those from WT mice (Fig. 2c). We further compared activation of downstream signaling molecules, Erk and Akt, in BM-derived macrophages (BMDMs) at day 2 when stimulated with M-CSF. M-CSF markedly stimulated phosphorylation of Erk and Akt in BMDMs from WT mice at 5 min (Fig. 2d). By contrast, there was significant decrease in protein phosphorylation of Erk and Akt in BMDMs from *Ndrg1* KO mice (Fig. 2d).

On the other hand, granulocyte–macrophage colony-stimulating factor (GM-CSF), also known as CSF2, is known to be required for the differentiation of monocyte-derived inflammatory DCs^22^,^23^. We then examined GM-CSF-induced differentiation of DCs by using BM cells as DC precursors. *Ndrg1* deficiency in BM cells significantly suppressed GM-CSF-induced differentiation into CD11c+ DCs (BMDCs) at days 3 and 5 (Fig. 2e). As compared with BMDCs at day 2 from WT mice, significant decrease in protein phosphorylation of STAT5 was observed in BMDCs from *Ndrg1* KO mice when stimulated with GM-CSF (Fig. 2f). Taken together, NDRG1 is required for the proliferation and differentiation of both BMDMs and BMDCs when stimulated with M-CSF or GM-CSF, respectively.

### NDRG1 deficiency induces abnormal bone formation by impairing the differentiation of macrophage lineage cells into osteoclasts

**Figure 3:**
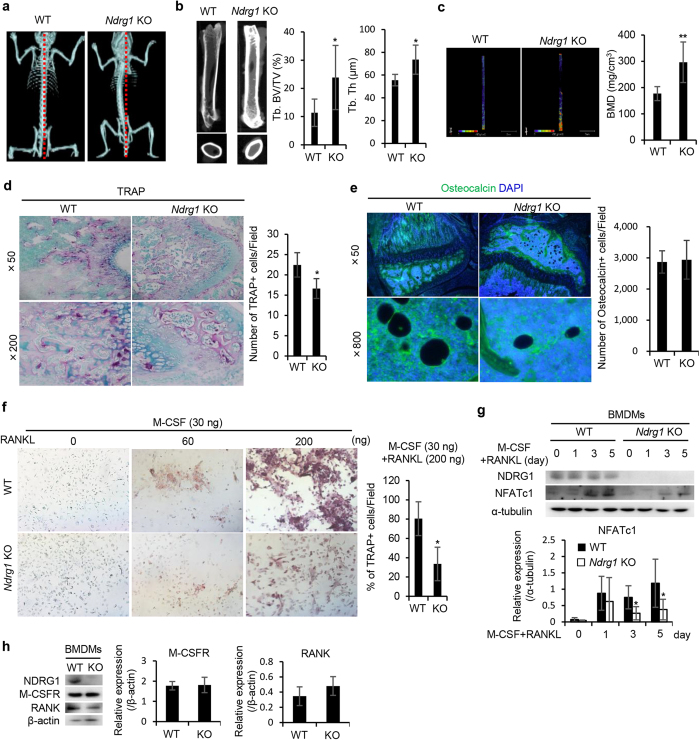
NDRG1 deficiency leads to abnormal bone formation caused by the impaired ability of macrophage lineage cells to differentiate into osteoclasts. (**a**) Comparison of bone formation between WT and *Ndrg1* KO mice at 3 month of age. Microcomputed tomography (μCT) analysis showed an abnormal curvature of the spine in *Ndrg1* KO mice. (**b**) Representative μCT images (left) and quantification of trabecular bone fraction (Tb. BV/TV) (middle) and trabecular thickness (Tb. Th) (right) (*n* = 4 per genotype). (**c**) Representative trabecular bone mass images (left) and quantification of bone mineral density (BMD) (right) (*n* = 6 per genotype). (**d**) Representative TRAP staining images (left) and quantitative analysis of the number of TRAP+ osteoclasts in the femoral diaphysis (right) (*n* = 4 per genotype). Original magnification ×50 (top) and ×200 (bottom). (**e**) Representative images of immunostaining of osteocalcin (green) (left) and quantification of the number of osteocalcin+ cells in the femoral diaphysis (right) (*n* = 4 per genotype). Original magnification ×50 (top) and ×800 (bottom). (**f**) Images of TRAP-stained BMDMs stimulated with M-CSF (30 ng/mL) and RANKL (60 ng/mL or 200 ng/mL) for 8 days to study the development of TRAP+ cells *in vitro* (left) (×40 original magnification). The percentages of TRAP+ cells (osteoclasts) are shown (right) (*n* = 3 per genotype). (**g**) Western blots analysis of NFATc1 expression in BMDMs from WT and *Ndrg1* KO mice at the indicated time points after stimulation with M-CSF (30 ng/mL) and RANKL (200 ng/mL). The lower panel shows the quantification of expression levels of NFATc1 normalized to the expression levels of α-tublin (*n* = 4 per genotype). Full-length blot are presented in Supplementary Figure 4. (**h**) Expression of M-CSF receptor (M-CSFR) and RANKL receptor (RANK) in BMDMs. Western blot analysis was performed with specific antibodies. The right panels show the quantification of expression levels of M-CSFR and RANK normalized to the expression levels of β-actin (*n* = 4 per genotype). Full-length blot are presented in Supplementary Figure 5. Each bar is an average ± SD, *P < 0.05; **P < 0.01 versus WT mice (two-tailed Student’s t-test).

Consistent with a previous study^9^, we first recognized a palpable curvature of the spine in *Ndrg1* KO mice at approximately 3 months after birth. Microcomputed tomography (μCT) analysis revealed abnormal curvature of the spine in *Ndrg1* KO mice (Fig. 3a). The trabecular bone volume (Tb. BV/TV) and thickness (Tb. Th) were higher in *Ndrg1* KO mice compared to WT mice (Fig. 3b). Furthermore, the bone mineral density (BMD) of the femur was higher in *Ndrg1* KO mice than in WT mice (Fig. 3c). Fewer tartrate-resistant acid phosphatase (TRAP)+ cells (osteoclasts) were found in the femurs of *Ndrg1* KO mice than in WT mice (Fig. 3d). In contrast, similar numbers of osteocalcin+ cells (osteoblasts) were found in *Ndrg1* KO mice and WT mice (Fig. 3e), indicating that *NDRG1* deficiency selectively affected osteoclasts and not osteoblasts.

To examine the role of NDRG1 in the differentiation of macrophages into osteoclasts, we compared the differentiation capacity of BMDMs into mature osteoclasts following stimulation with M-CSF (30 ng/mL) and receptor activator of NF-κB ligand (RANKL) (60 ng/mL or 200 ng/mL) *in vitro*. After BMDMs derived from WT mice were incubated with M-CSF and 200 ng/mL RANKL for 8 days, approximately 80% of the cells became TRAP+ cells (Fig. 3f). However, less than 40% of the BMDMs derived from *Ndrg1* KO mice became TRAP+ cells (Fig. 3f). Nuclear factor of activated T cells c1 (NFATc1) is a master regulator of RANKL-induced osteoclastogenesis through calcium signaling pathway^24^. We further examined NFATc1 expression in BMDMs when stimulated with M-CSF and RANKL. NFATc1 expression was augmented in BMDMs from both mice at days 3 and 5 after stimulation of M-CSF (30 ng/mL) and RANKL (200 ng/mL), but there was significant decrease in protein induction of NFATc1 in BMDMs from *Ndrg1* KO mice (Fig. 3g). Taken together, these results indicate that *NDRG1* deficiency impaired the differentiation of macrophage lineage cells into osteoclasts. The expression levels of the M-CSF receptor (M-CSFR) and RANKL receptor (RANK) in BMDMs were similar in WT and *Ndrg1* KO mice (Fig. 3h).

### Tumor growth and angiogenesis are also impaired in *Ndrg1* KO mice

**Figure 4:**
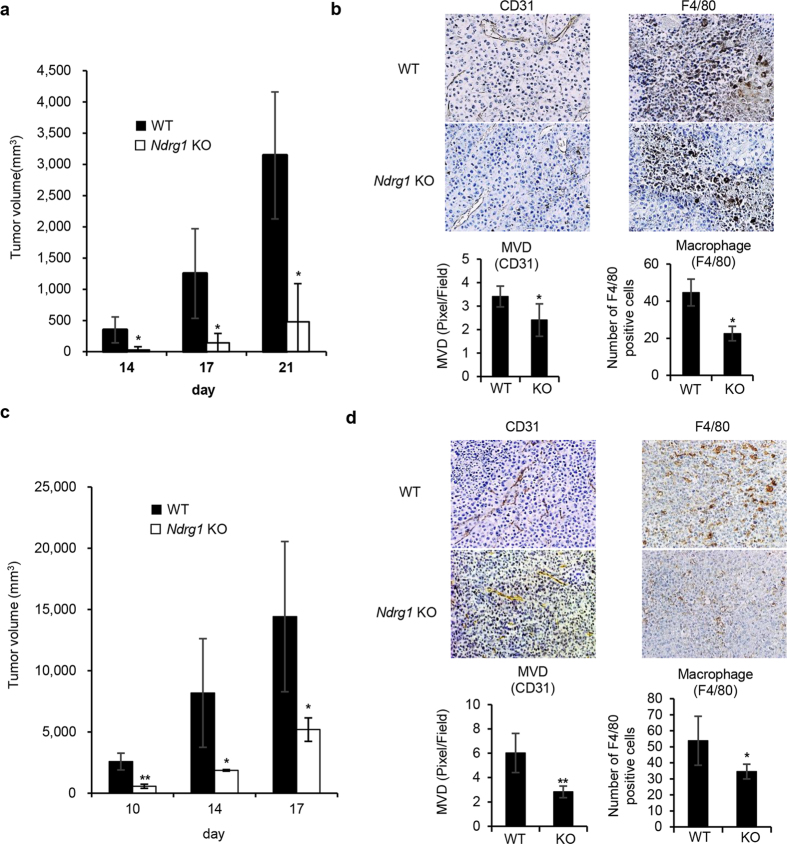
Tumor growth, tumor angiogenesis and macrophage infiltration. (**a**) Growth of B16/BL6 tumors in male WT and *Ndrg1* KO mice. Tumor growth rates were assessed after mice were subcutaneously inoculated with B16/BL6 melanoma cells at day 0 (*n* = 4 per genotype). (**b**) Angiogenesis and infiltrating macrophages in B16/BL6 tumors at day 21 were assessed by IHC using antibodies for vascular endothelium (CD31) (left) and macrophages (F4/80) (right). B16/BL6 tumors were quantitatively analyzed by scoring five areas in each tumor section for microvascular density (MVD) and F4/80-positive cells (*n* = 4 per genotype). Original magnifications ×100 (left) and ×200 (right). (**c**) Growth of LLC/3LL tumor in male WT and *Ndrg1* KO mice. Tumor growth rates were assessed after mice were subcutaneously inoculated with LLC/3LL cells at day 0 (*n* = 4 per genotype). (**d**) Angiogenesis and infiltrating macrophages in LLC/3LL tumors were assessed at day 17 by IHC using antibodies for vascular endothelium (CD31) (left) and macrophages (F4/80) (right). LLC/3LL tumors were quantitatively analyzed at day 17 by scoring five areas in each tumor section for microvascular density (MVD) and F4/80-positive cells (*n* = 4 per genotype). Original magnifications ×100 (left) and ×200 (right). Each bar is an average ± SD, *P < 0.05; **P < 0.01 versus WT mice (two-tailed Student’s t-test).

To examine whether *NDRG1* knockdown affects tumor growth and angiogenesis, we compared tumor growth and angiogenesis between WT and *Ndrg1* KO mice in a syngeneic mouse tumor model. Tumors resulting from subcutaneously implanted B16/BL6 cells showed significantly (*P < 0.05) slower growth rates in *Ndrg1* KO mice than in WT mice (Fig. 4a). Thin sections of these tumors taken at day 21 after implantation displayed significantly (*P < 0.05) decreased tumor neovascularization (CD31+, MVD) and infiltration of F4/80+ macrophages (Fig. 4b). We also compared tumor growth rates and angiogenesis using LLC/3LL mouse lung cancer cells. The growth rates of the LLC/3LL tumors were also significantly (**P < 0.01, *P < 0.05) slower in *Ndrg1* KO mice than in WT mice (Fig. 4c). Tumor angiogenesis and the number of infiltrating macrophages in the tumors were also found to be significantly (**P < 0.01, *P < 0.05) reduced in *Ndrg1* KO mice compared to WT mice (Fig. 4d). Together, these results suggest that NDRG1 promotes tumor growth and angiogenesis, which is accompanied by the infiltration of macrophages into the tumor.

### NDRG1 deficiency in tumor-associated macrophages impairs the production of angiogenic factors and M1/M2 phenotype-specific biomarkers

**Figure 5:**
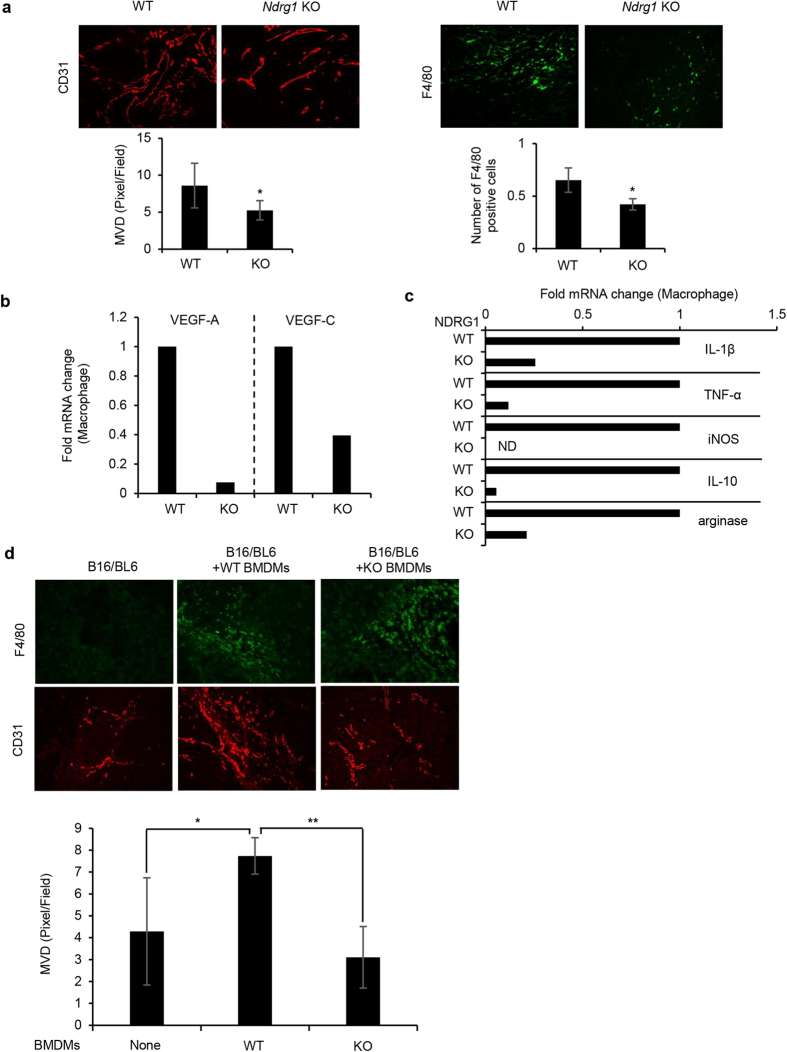
Evaluation of VEGF-A, VEGF-C, and M1- and M2-specific biomarkers in tumor-associated macrophages in Matrigel plugs in WT and *Ndrg1* KO mice. (**a**) Tumor angiogenesis and macrophage infiltration in Matrigel plugs containing B16/BL6 cells. Tumor angiogenesis (MVD) and infiltrating macrophages (F4/80+) were determined by IHC analysis of Matrigel plugs using CD31 (left) and F4/80 (right) antibodies (top). Matrigel plugs were quantitatively analyzed at day 7 by scoring five areas in each section for microvascular density (MVD) and F4/80-positive cells (bottom) (*n* = 5 per genotype). (**b**) *Vegf-A* and *Vegf-C* expression in macrophages isolated from Matrigel plugs was determined by qRT-PCR. The data were normalized by the WT expression levels of each factor. (**c**) Expression of M1-type (IL-1β, TNF-α and iNOS)- and M2-type (IL-10 and arginase)-specific biomarkers in macrophages isolated from Matrigel plugs was determined by qRT-PCR. The data were normalized by the WT expression levels of each factor. ND; not detectable. (**d**) Reduction of tumor angiogenesis in BM-eradicated male WT mice following the transfer of BMDMs from *Ndrg1* KO mice. Matrigel plugs containing B16/BL6 cells (1 × 10^5^ cells per plug) were subcutaneously inoculated with BMDMs (1 × 10^6^ cells) derived from WT or *Ndrg1* KO mice at day 0. Tumor angiogenesis (MVD) and infiltrating macrophages (F4/80+) were evaluated by IHC analysis of Matrigel plugs using CD31 (red) and F4/80 (green) antibodies (top). Matrigel plugs at day 7 were quantitatively analyzed by scoring five areas in each section for microvascular density (MVD) (bottom) (*n* = 4 per genotype). Each bar is an average ± SD, *P < 0.05; **P < 0.01 versus WT mice (two-tailed Student’s t-test). Original magnification × 200 for all panels.

We further examined whether tumor-associated macrophages could produce potent angiogenic factors as well as whether they have M1- and/or M2-specific characteristics in *Ndrg1* KO mice. We performed Matrigel plug assays *in vivo*. Neovasculature developed within the Matrigel-associated cancer cells on day 7 to 10^17^,^25^. We observed an increased number of CD31+ neovessels and F4/80+ infiltrating macrophages in the Matrigel plugs at day 7, but smaller numbers of both cell types were found in *Ndrg1* KO mice than in WT mice (Fig. 5a, top panel). The microvascular density (MVD) and number of infiltrating macrophages were also significantly (*P < 0.05) decreased in *Ndrg1* KO mice compared to WT mice (Fig. 5a). Further analysis showed that the expression levels of *Vegf-A* and *Vegf-C* in macrophages in *Ndrg1* KO mice were approximately 30% or less those in WT mice (Fig. 5b).

Activated tumor-associated macrophages are classified into M1-type and M2-type macrophages^19^,^20^. We characterized the macrophages that infiltrated into the Matrigel plugs using specific markers for the M1-type (IL-1β, TNF-α and inducible nitric oxide synthase [iNOS]) and M2-type (IL-10 and arginase) cells. The expression of all tested M1- and M2-specific markers was markedly reduced in macrophages in *Ndrg1* KO mice compared to WT mice (Fig. 5c). These data indicate that decreased tumor angiogenesis and growth in *Ndrg1* KO mice may be attributable to the impaired potential of inactive macrophages to transform into functionally active macrophages, including angiogenic macrophages as well as into both M1- and M2-type macrophages.

In the Matrigel plug assays, we observed more abundant tumor angiogenesis when both BMDMs from WT mice and cancer cells were implanted into BM-eradicated WT mice than when only cancer cells were implanted (Fig. 5d). However, we did not observe any increased tumor angiogenesis when both BMDMs from *Ndrg1* KO mice and cancer cells were implanted (Fig. 5d). These findings suggest that *NDRG1* deficiency in macrophages specifically impairs tumor angiogenesis.

### NDRG1 deficiency impairs macrophage activity *in vitro*

**Figure 6:**
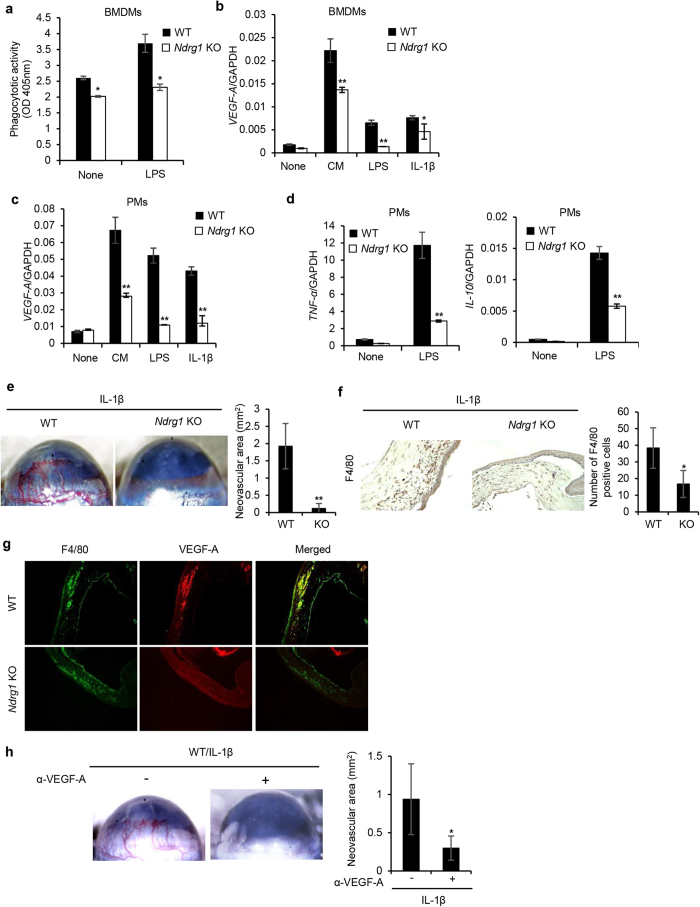
NDRG1 deficiency impairs functions of macrophages and inflammatory angiogenesis. (**a**) Comparison of LPS-stimulated phagocytotic activity of BMDMs between WT and *Ndrg1* KO mice (*n* = 3 per genotype). (**b,c**) CM-, LPS-, or IL-1β-stimulated *Vegf-A* expression in BMDMs (**b**) and peritoneal macrophages (PMs) (**c**) from WT and *Ndrg1* KO mice. Macrophages were treated with CM, LPS, and IL-1β, and mRNA levels of *Vegf-A* were determined by qRT-PCR (*n* = 3 per genotype). (**d**) Expression of M1-type (TNF-α)- and M2-type (IL-10)-specific biomarkers in PMs stimulated with LPS for 4 h was determined by qRT-PCR (*n* = 3 per genotype). (**e**) Corneal neovascularization induced by IL-1β. Photos showing neovascularization in the corneas of male WT and *Ndrg1* KO mice 7 days after Hydron pellets containing IL-1β (30 ng) were implanted into the corneas (left) (original magnification ×20). Quantitative analysis of neovascularization on day 7 (right) (*n* = 5 per genotype). Areas are expressed in mm^2^. (**f**) IHC analysis of corneas treated with IL-1β (day 7) was performed using a macrophage-specific antibody (F4/80) (left) (original magnification ×200). The number of F4/80-positive cells that infiltrated into the corneas was quantified (right) (*n* = 5 per genotype). (**g**) IHC analysis of IL-1β-treated corneas (day 7) using a macrophage-specific antibody (F4/80) and a VEGF-A-specific antibody. F4/80+ macrophages (green) costained with VEGF-A (red), as shown by yellow (merged) (original magnification × 100). (**h**) Inhibition of IL-1β-induced angiogenesis by anti-VEGF-A neutralizing antibodies (α-VEGF-A) (left) (original magnification × 20). Quantitative analysis of neovascularization on day 7 (right) (*n* = 3 per genotype). Areas are expressed in mm^2^. Each bar is an average ± SD, *P < 0.05; **P < 0.01 (two-tailed Student’s t-test).

We next examined whether *NDRG1* deficiency could directly affect phagocytotic activity and expression of *Vegf-A, Tnf-α*, and *Il-10* under various conditions (Fig. 6a–d). BMDMs derived from *Ndrg1* KO mice showed lower phagocytotic activity under both basal and LPS-stimulated conditions than BMDMs from WT mice (Fig. 6a, *P < 0.05). We previously reported that the expression of the potent angiogenic factor VEGF-A in macrophages was enhanced by various inflammatory stimuli^26^,^27^,^28^. The expression of *Vegf-A* was markedly increased by conditioned medium (CM) from B16/BL6 cells, and moderately increased by LPS and IL-1β in BMDMs from both WT and *Ndrg1* KO mice, but the stimulatory effects were much smaller in BMDMs from *Ndrg1* KO mice (Fig. 6b, **P < 0.01, *P < 0.05).

The expression of *Vegf-A* was also markedly increased by CM, LPS and IL-1β in peritoneal macrophages (PMs) from both WT and *Ndrg1* KO mice; however, the enhanced expression levels of *Vegf-A* in cells derived from *Ndrg1* KO mice were less of those in cells derived from WT mice (Fig. 6c, **P < 0.01). Furthermore, the expression of specific markers for M1-type (TNF-α) and M2-type (IL-10) macrophages was markedly increased by LPS in PMs from both WT and *Ndrg1* KO mice, but the stimulatory effects were approximately 60% or less in cells from *Ndrg1* KO mice (Fig. 6d, **P < 0.01).

### *Ndrg1* KO mice exhibit poor IL-1β-induced angiogenesis accompanied by decreased infiltration of VEGF-A-producing macrophages

We have previously reported that the inflammatory cytokine IL-1β induces angiogenesis in corneas via the infiltration of activated macrophages that express potent angiogenic factors such as VEGF-A, VEGF-C and VEGF-D^29^,^30^. To examine whether *NDRG1* deficiency affects IL-1β-induced angiogenesis, we first compared angiogenesis in response to IL-1β by corneal micropocket assay. IL-1β induced angiogenesis in WT mice, but only slightly, if at all, in *Ndrg1* KO mice (Fig. 6e). More F4/80+ macrophages infiltrated the cornea in WT mice than in *Ndrg1* KO mice following IL-1β stimulation (Fig. 6f). Quantitative analysis confirmed that the macrophage infiltration induced by IL-1β was significantly decreased in *Ndrg1* KO mice (Fig. 6f). Furthermore, we examined whether infiltrated macrophages in IL-1β-treated corneas expressed VEGF-A. IHC analysis with a macrophage-specific antibody (F4/80) and VEGF-A-specific antibodies showed that F4/80+ macrophages (green) co-stained with VEGF-A (red) in the corneas of WT mice but not in *Ndrg1* KO mice (Fig. 6g). The administration of a neutralizing antibody against VEGF-A almost completely blocked IL-1β-induced angiogenesis in WT mice (Fig. 6h). Taken together, these findings suggested that IL-1β-induced poor angiogenesis was attributable to the impaired VEGF-A-producing potential of macrophages in *Ndrg1* KO mice.

## Discussion

**Figure 7:**
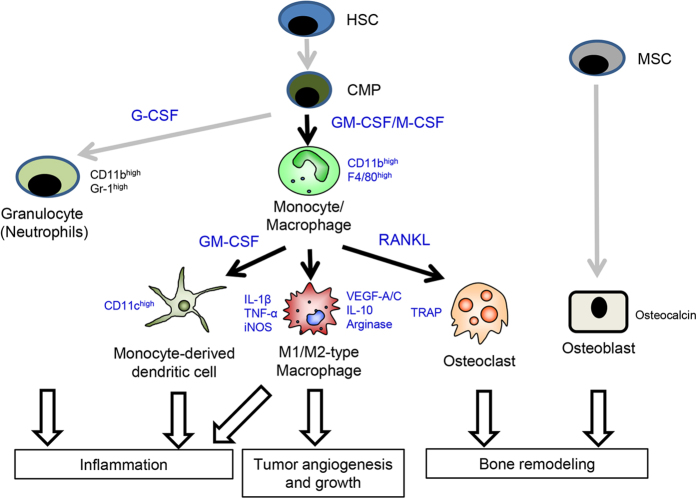
A model showing how NDRG1 plays its essential roles in promotion of bone remodeling and tumor growth/angiogenesis. NDRG1 promotes M-CSF-induced proliferation and differentiation of hematopoietic stem cells (HSCs)/common myeloid precursors (CMPs), leading to an increase in the population of monocytes/macrophages but not granulocytes. NDRG1 further promotes the terminal differentiation of monocytes/macrophages into M1- and M2-type macrophages, osteoclasts, and monocyte-derived dendritic cells. NDRG1, however, does not play a critical role in the differentiation of mesenchymal stem cells (MSCs) into osteoblasts. Black arrows, NDRG1 is involved; gray arrows, NDRG1 is not involved.

This study provides the first demonstration that NDRG1 is required for the differentiation of macrophage lineage cells, supporting bone remodeling and pathological angiogenesis (Fig. 7). We revealed a novel role of NDRG1 in the differentiation processes from macrophage progenitor cells (hematopoietic stem cells [HSCs]/common myeloid precursors [CMPs]) into monocytes/macrophages/DCs when stimulated with M-CSF or GM-CSF. M-CSF and GM-CSF are well-known primary growth factors that regulate the survival, proliferation and differentiation of macrophages, DCs and other mononuclear phagocytotic lineage cells^21^. RANKL is also well-known protein that promotes the differentiation of monocytes/macrophages into osteoclasts when combined with M-CSF (Fig. 7). Osteoclasts are the exclusive bone-resorptive cells and play an essential role in physiological bone formation and homeostatic bone remodeling. During bone formation and remodeling, osteoclast bone resorption is closely harmonized with osteoblast bone formation^31^,^32^. An imbalance of osteoclasts and osteoblasts leads to either osteopetrosis or excessive bone resorption. This study also demonstrated that *NDRG1* deficiency induced this imbalance and increased bone volume, akin to symptoms of osteopetrosis^33^, through selectively interference with the generation and function of osteoclasts, but not osteoblasts. These data suggested that *NDRG1* may be a newly responsible gene for this disease. On the other hand, the abnormal curvature of the spine is often due to neuromuscular dysfunction, however its responsible genes are not fully understood^34^. NDRG1 knockdown causes a demyelinating sensory in mice and humans^8^,^9^. It is therefore possible that the abnormal curvature of the spine in *Ndrg1* KO mice may be rather more attributable to the neuropathy than the primary disorder of osteoclasts. Further study should be required to determine the cause of the abnormal curvature of the spine by NDRG1 knockdown.

*NDRG1* deficiency did not affect the expression of the receptors for M-CSF and RANKL, indicating that altered post-receptor signaling was responsible for the impaired differentiation of osteoclasts in *Ndrg1* KO mice. The activation of NFAT is mediated by a specific phosphatase, calcineurin, which is activated by calcium-calmodulin signaling. The *NFATc1* promoter contains NFAT-binding sites, and NFATc1 specifically autoregulates its own promoter during osteoclastogenesis^24^. Calcium signaling is essential for the differentiation of cells into mature osteoclasts in response to M-CSF and RANKL^35^. On the other hand, calcium ions and related cellular signaling are closely associated with the expression and function of NDRG1^1^. Thus, NDRG1 may play an essential role in the differentiation of macrophage lineage cells into osteoclasts by regulating calcium signaling. Further study should be required to determine whether NDRG1 modulates activation of calcium signaling pathway, such as calcineurin and calmodulin, during osteoclastogenesis.

A recent discovery in tumor biology is that the tumor microenvironment plays an essential role in the malignant progression of cancer cells, supporting the novel concept that cancer cells cannot function alone. In particular, monocytes/macrophages are transformed into tumor-associated macrophages when exposed to a tumor environment^19^,^36^,^37^,^38^. We have reported that the augmented expression of IL-1α, IL-1β, VEGF-A and VEGF-C by macrophages promotes tumor growth and tumor angiogenesis/lymphangiogenesis^25^. These tumor-associated macrophages are mainly composed of M2-type macrophages, suggesting the preferential involvement of M2-type macrophages over the M1-type in tumor angiogenesis and lymphangiogenesis^25^. Furthermore, NDRG1 is highly expressed in macrophages in the tumor microenvironment^39^,^40^. In this study, we demonstrated the involvement of NDRG1 in tumor progression, especially tumor angiogenesis, through enhancing the differentiation of tumor-associated macrophages (Fig. 7). However, which molecules are specifically regulated by NDRG1 during differentiation processes remains unclear.

In conclusion, *NDRG1* deficiency in a host induces an imbalance in the differentiation of macrophage lineage cells, which impairs both bone remodeling and pathological angiogenesis. NDRG1 may contribute to the homeostatic balance of both bone remodeling and angiogenesis through its regulatory role in the differentiation of macrophage lineage cells. NDRG1 may also have potentially important implications for the diagnosis for osteodystrophy and cancer patients. Increased understanding of NDRG1 and its downstream regulatory pathway is required to establish the translational potential of NDRG1 in therapeutic strategies.

## Methods

### Reagents and antibodies

Polyclonal antibody against full-length Ndrg1 was a kindly provided from Dr. Kokame K. (National Cerebral and Cardiovascular Center Research Institute, Osaka, Japan). Anti-mouse CD31 (1:70; ER-MP12; T-2001) antibody was purchased from BMA BIOMEDICALS (Augst, Switzerland); anti-mouse F4/80 (1:200; Cl:A3-1; MCA497R) antibody was from AbD Serotec (Raleigh, NC); anti-mouse vascular endothelial growth factor (VEGF)-A (1:100; sc-507), and anti- nuclear factor of activated T cells c1 (NFATc1) (1:1000; H-110; sc-13033) antibodies were from Santa Cruz Biotechnology (Dallas, TX); CF488 conjugated anti-rat IgG (1:500; 20027-1) and CF594 conjugated anti-rabbit IgG (1:500; 20152-1) antibodies were from Biotium (Hayward, CA); PE-conjugated anti-mouse CD11b (1:100; M1/70; 12-0112-82), and FITC-conjugated anti-mouse F4/80 (1:100; BM8; 11-4801-82) antibodies were from eBiosciences (San Diego, CA); FITC-conjugated anti-mouse Gr-1 (1:100; 1A8; 551460) antibody was from CALTAG LABORATORIES (Burlingame, CA); anti-β-actin (1:5000; ab8226) antibody was from Abcam (Cambridge, UK); anti-Erk 1/2 (1:1000; 9102), anti-phospho Erk 1/2 (1:1000; E10; 9106), anti-Akt (1:1000; 9272), anti-phospho Akt (Ser473) (1;1000; D9E; 4060), anti- signal transducer and activator of transcription5 (STAT5) (1:1000; 9363), anti-phospho STAT5 (Y694) (1:1000; D47E7; 4322), anti- macrophage colony-stimulating factor receptor (M-CSFR) (1:1000; 3152) antibodies were from Cell Signaling Technology (Beverly, MA); anti-receptor activator of NF-κB (RANK) (1:1000; 9A725; IMG-128A) antibody was from Novus Biologicals (Littleton, CO); anti-glyceraldehyde-3-phosphate dehydrogenase (GAPDH) (1:5,000; 2275-PC-100) antibody was from Trevigen (Gaithersburg, MD); anti-α-tubulin(1:5,000; B-5-1-2; T6074) antibody was from Sigma-Aldrich (St Louis, MO); PE-conjugated anti-mouse CD11c (1:100; HL3; 227401) was from BD Pharmingen (Franklin Lakes, NJ); mouse M-CSF and human interleukin (IL)-1β were from R&D system Inc. (Minneapolis, MN); mouse granulocyte macrophage colony-stimulating factor (GM-CSF) and mouse RANK ligand (RANKL) were from PeproTech, Inc. (Rocky Hill, NJ).

### Mice

The *Ndrg1* KO (−/−) mice on a C57BL6 background were purchased from Laboratory Animal Resource Bank, National Institutes of Biomedical Innovation, Health and Nutrition (Osaka, Japan)^9^. All our experiments compared *Ndrg1* KO mice with age- and gender-matched *Ndrg1* WT mice. All mice were housed in microisolator cages maintained under a 12-hr light/dark cycle. Water and food were supplied ad libitum. Animals were observed for signs of tumor growth, activity, feeding, and pain in accordance with the guidelines of the Harvard Medical Area Standing Committee on Animals. All animal experimental procedures in this study were reviewed and approved by the Animal Ethics Committee of Kyushu University, Fukuoka, Japan (Registration numbers: A26-018-1).

### Polymerase chain reaction (PCR) analysis

PCR analysis of DNA isolated from cut tail with primers; P1: AGCAGGCTCTTAAAGCGGCTCC, P2: CCGCCTCTGTCAAATTAGTAGCTG, and P3: GGGAGAGCTGAAGGCTGTTCTAGG. The product of P2 + P3 gives a wild-type band of 201 bp. A KO band of 268 bp is produced by primers P1 + P3. PCR was done in a final volume of 20 μL using Takara Ex Taq (Takara Bio, Shiga, Japan). Initial denaturing was done at 94 °C for 2 min followed by 35 cycles (94 °C for 30 s, 53 °C for 30 s, 72 °C for 2 min) and final extension at 72 °C for 5 min.

### Multiplex suspension array

Blood plasma was collected from 5 week old male mice. The concentration of cytokines in the blood plasma was quantified using a Multiplex suspension array (GeneticLab Co., Ltd., Hokkaido, Japan) according to the manufacturer’s instructions.

### Preparation of whole blood cells

Whole blood was collected from 5–10 week old male WT and *Ndrg1* KO mice. The blood was then treated with red blood cell lysis buffer (0.83% NH_4_Cl) for 5 min at room temperature. After a phosphate buffered saline (PBS) wash, the cells were used for the following experiments.

### Flow cytometry

Whole blood cells were suspended with antibody diluent (Dako, Glostrup, Denmark) and were incubated with anti-CD11b and anti-F4/80, or anti-CD11b and anti-Gr-1 antibody for 20 min. BM derived macrophages (BMDMs) or BM derived dendritic cells (BMDCs) were suspended with antibody diluent (Dako) and were incubated with anti-CD11b and anti-F4/80, or anti-CD11c antibody for 30 min. After twice washes with PBS, data were acquired using a FACS Calibur system (Becton-Dickinson, Moutain View, CA). The data were analyzed using the Cell Quest software program (BD Biosciences).

### BM cell proliferation assay

BM cells were obtained by flushing mouse tibiae and femurs from 5–10 week old male WT and *Ndrg1* KO mice with ice-cold PBS and passing the suspension through a cell strainer with a 70 μm cut-off. For BM cell proliferation assay, cells were seeded at 2 × 10^4 ^cells in DMEM containing 10% FBS and M-CSF (20 ng/mL). The number of cells was counted on days 1, 3, 5 and 7. The medium was changed every 2 days. For the analysis of M-CSF-induced BM cell proliferation assay, cells were seeded at 2 × 10^4^ cells in DMEM containing 10% FBS and M-CSF (20 ng/mL) for 2 days. Then, the medium was changed to DMEM containing 2% FBS, and incubated for 24 h. Then various concentration of M-CSF was added, followed by incubation for 3 days and the number of cells was counted.

### Preparation of BMDMs and BMDCs

BM cells were obtained by flushing mouse tibiae and femurs from male WT and *Ndrg1* KO mice with ice-cold PBS and passing the suspension through a cell strainer with a 70 μm cut-off. Three hours after cell seeding in culture dishes, cells were washed twice. For preparation of BMDMs, adherent cells were incubated in Dulbecco’s Modified Eagle’s Medium (DMEM) medium supplemented with 10% fetal bovine serum (FBS) and 20 ng/mL M-CSF (R&D, Minneapolis, MN) at 37 °C for 2–7 days. BMDCs were also obtained by flushing mouse tibiae and femurs from male WT and *Ndrg1* KO mice with ice-cold PBS and passing the suspension through a cell strainer with a 70 μm cut-off. Cells were then treated with red blood cell lysis buffer (0.83% NH_4_Cl) for 5 min at room temperature. After twice PBS washes, BM cells were cultured RPMI 1640 medium supplemented with 10% FBS, 50 mM 2-mercaptoethanol (Sigma-Aldrich, St Louis, MO), and 25 ng/mL GM-CSF (PeproTech, Rocky Hill, NJ) for 2–8 days.

### *In vitro* stimulation assay in BMDMs and BMDCs

BMDMs or BMDCs at day 2 were incubated with serum free DMEM or RPMI medium for 6 h. They were then stimulated with M-CSF (30 ng/mL) or GM-CSF (10 ng/mL). For the expression analysis of NFATc1, BMDMs at day 7 were incubated with 10% FBS DMEM supplemented with M-CSF (30 ng/mL) and RANKL (200 ng/mL). At the indicated time points, cells were harvested and subjected to SDS-PAGE.

### Microcomputed tomography (μCT) analysis

Femurs were resected from 3 month old male mice, and analyzed by high-resolution μCT (R_mCT, Rigaku, Tokyo, Japan). CT scans were performed at a voltage of 60 kV, a current of 60 μA and a resolution of 50 μm per pixel. Trabecular bone structure was analyzed using a 3D image analysis system (TRI/3D-BON; RATOC System Engineering, Tokyo, Japan). We established cross-sectional images of the femurs to perform two-dimensional morphometric analyses of the cortical bone and three-dimensional histomorphometric analysis of the trabecular bone. Parameters were calculated in 3D as follows: trabecular volumetric bone mineral density (vBMD) was determined using a reference phantom (KYOTO KAGAKU, Kyoto, Japan). Using a vBMD value for trabecular bone of >100 mg/cm^3^, the BM was extracted, and the Tb. BV/TV and Tb. Th were analyzed.

### Matrigel plug assay

Six–ten week old male mice were injected subcutaneously at the abdominal midline with 0.5 mL of growth factor-reduced Matrigel matrix (BD Biosciences) supplemented with B16/BL6 cells (1 × 10^5 ^cells) as previously described^25^. To analyze BM suppression, 6–10 week old male WT recipient mice were exposed to 3-Gy, sublethal whole-body irradiation to suppress BM and temporarily deplete it of leukocytes. Seven days after the irradiation, the mice were injected subcutaneously with 0.5 mL of growth factor-reduced Matrigel matrix containing B16/BL6 cells (1 × 10^5 ^cells), with or without BMDMs (5 × 10^5^ cells) that were derived from 6–10 week old male WT or *Ndrg1* KO mice. After 7 days, the Matrigel plugs were removed and snap frozen in optimal cutting temperature (OCT) compound. The tissue blocks were cut into 5 μm sections, which were first air dried and then fixed for 3 min in cold acetone. The sections were stained with anti-CD31 and anti-F4/80 antibodies.

### Preparation of peritoneal macrophages (PMs)

Peritoneal macrophages were obtained from intraperitoneal injection of 4 mL of PBS into 6–10 week old male mice. The cells were suspended in DMEM medium and incubated for 120 min at 37 °C in a CO_2_ incubator to allow them to adhere to the plates. The medium was then withdrawn and non-adherent cells were removed by washing the plates twice with pre-warmed PBS.

### *In vitro* macrophage stimulation assay

PMs and BMDMs were incubated with DMEM supplemented with 2% FBS at 37 °C for 24 h, then stimulated with Lipopolysaccharide (LPS) (1 μg/mL) for 4 h, IL-1β (1 ng/mL) for 6 h, or B16/BL6 CM for 24 h, and mRNA was collected using ISOGEN reagent.

### Statistical analysis

All results are expressed as mean ± SD of *n* observations, and statistical differences among the groups were assessed by two-tailed Student’s *t*-test. A *P* value of less than 0.05 was considered significant.

## Additional Information

**How to cite this article**: Watari, K. *et al*. Impaired differentiation of macrophage lineage cells attenuates bone remodeling and inflammatory angiogenesis in *Ndrg1* deficient mice. *Sci. Rep.* **6**, 19470; doi: 10.1038/srep19470 (2016).

## Supplementary Material

Supplementary Information
