# Supplementary material for: Impaired differentiation of macrophage lineage cells attenuates bone remodeling and inflammatory angiogenesis in Ndrg1 deficient mice
Source: Sci Rep. 2016 Jan 18;6:19470. doi: 10.1038/srep19470 (PMC4726041; doi:10.1038/srep19470)
Supplement: Supplementary Information [file srep19470-s1.pdf]

Supplementary Information

Impaired differentiation of macrophage lineage cells attenuates bone remodeling and inflammatory angiogenesis in *Ndrg1* deficient mice

Kosuke Watari[1], Tomohiro Shibata[1], Hiroshi Nabeshima[1], Ai Shinoda[1], Yuichi Fukunaga[1], Akihiko Kawahara[2], Kazuyuki Karasuyama[3], Jun-ichi Fukushi[3], Yukihide Iwamoto[3], Michihiko Kuwano[4], and Mayumi Ono[1]*

[1]Department of Pharmaceutical Oncology, Graduate School of Pharmaceutical Sciences, Kyushu University, Fukuoka 812-8582, Japan

[2]Department of Diagnostic Pathology, Kurume University Hospital, Kurume 830-0011, Japan

[3]Department of Orthopedic Surgery, Graduate School of Medical Sciences, Kyushu University, Fukuoka 812-8582, Japan

[4]Cancer Translational Research Center, St. Mary's Institute of Health Sciences, St. Mary's Hospital, Kurume 830-8543, Japan

***Corresponding Author**:

Mayumi Ono, Ph.D.,

Department of Pharmaceutical Oncology, Graduate School of Pharmaceutical Sciences, Kyushu University

3-1-1 Maidashi, Higashi-ku, Fukuoka 812-8582, Japan;

Phone and Fax: +81-92-642-6296

E-mail: mono@phar.kyushu-u.ac.jp

NDRG1 **α-tubulin**

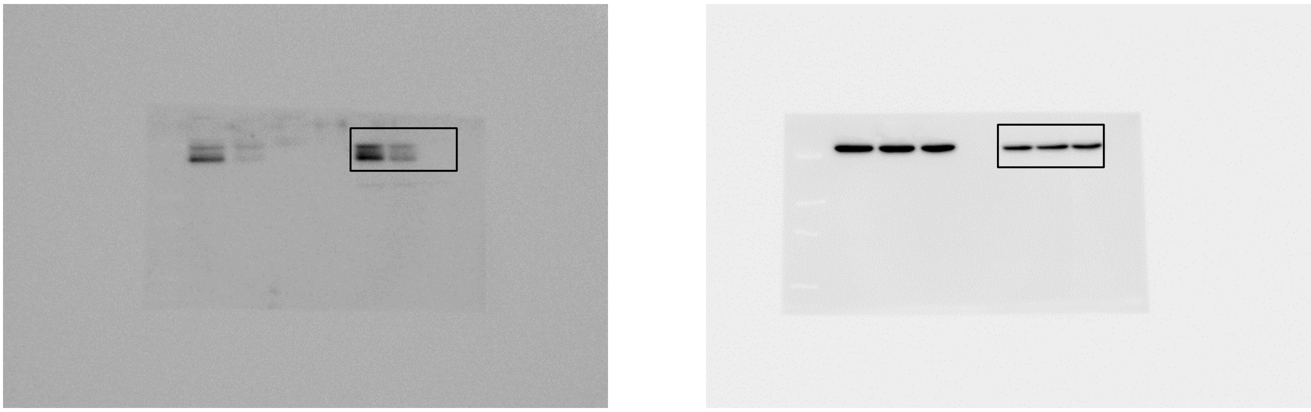

Figure S1. Presentation of original immunoblot shown in Figure 1a.
The cropped parts of immunoblot were indicated with black boxes.

NDRG1

GAPDH

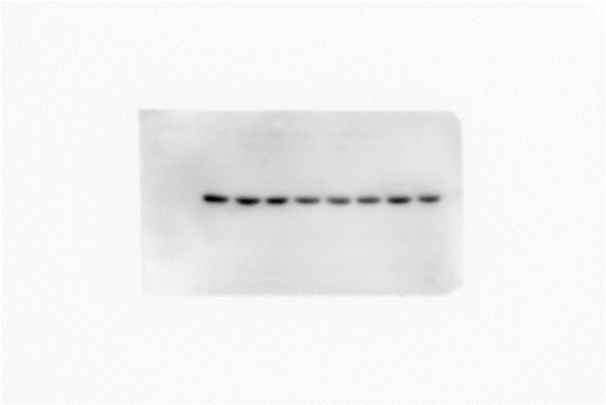

p-Akt

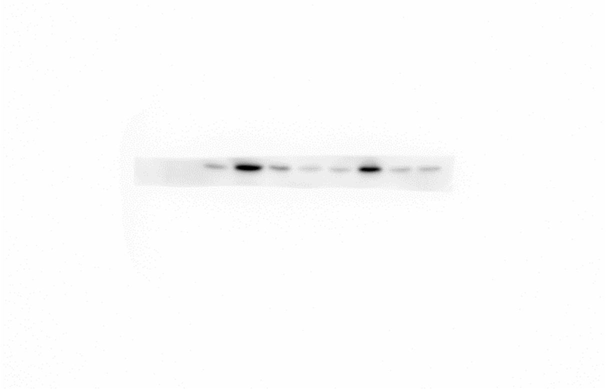

Akt

p-Erk

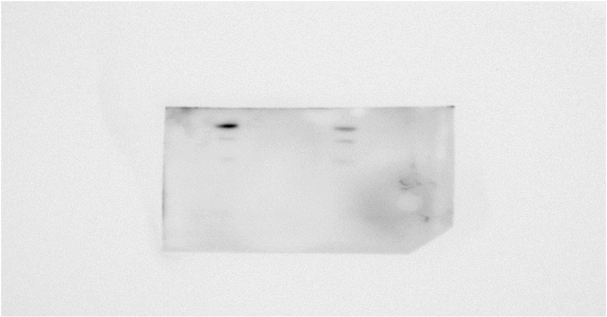

Erk

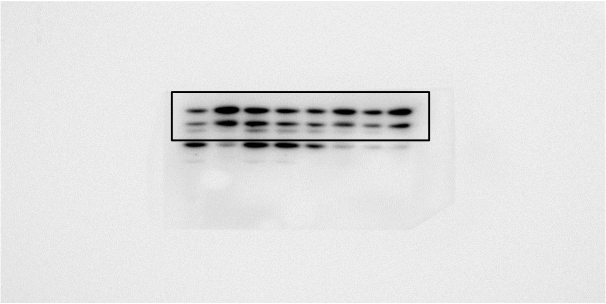

Figure S2. Presentation of original immunoblot shown in Figure 2d. The cropped parts of immunoblot were indicated with black boxes.

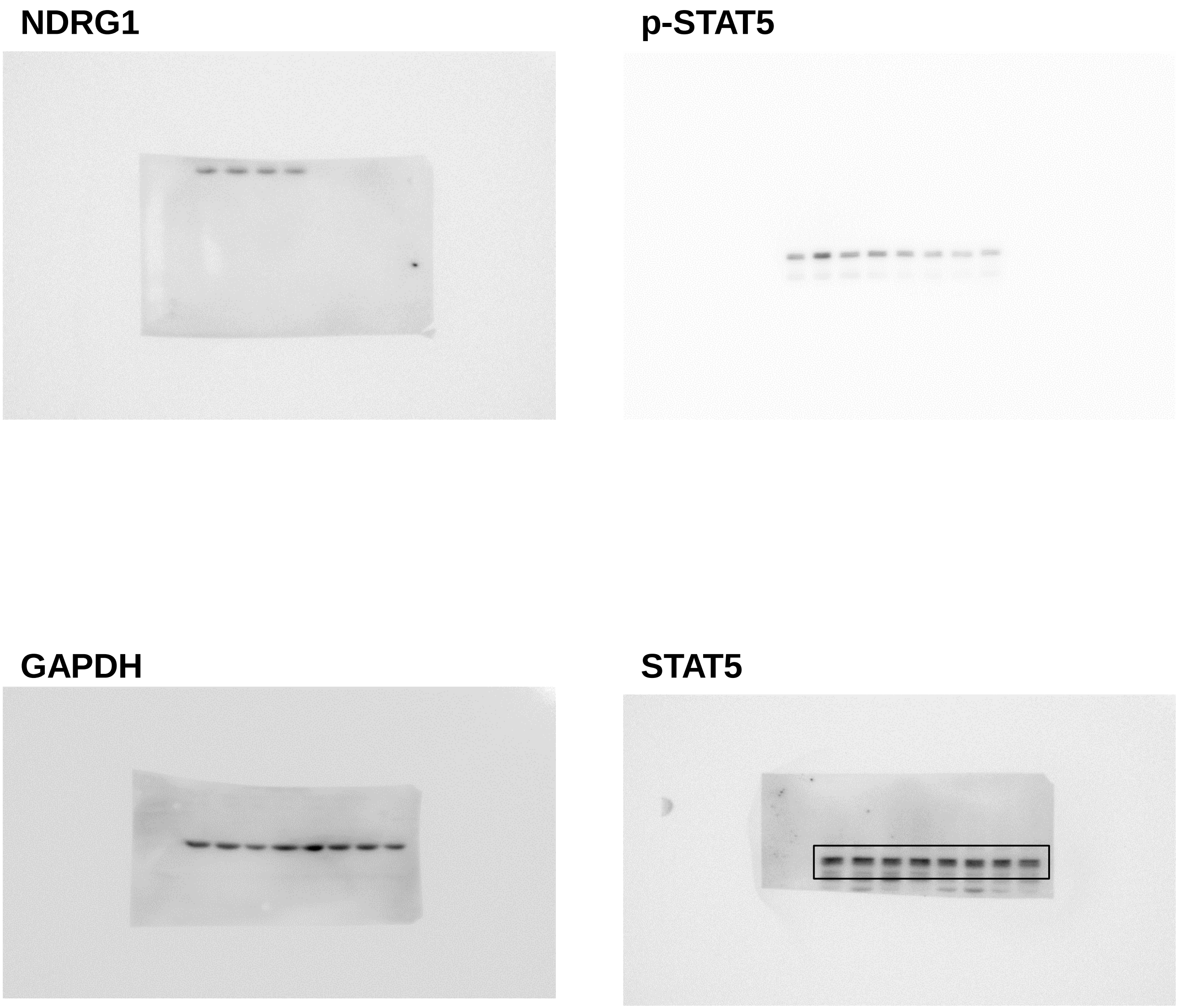

Figure S3. Presentation of original immunoblot shown in Figure 2f.
The cropped parts of immunoblot were indicated with black boxes.

NDRG1

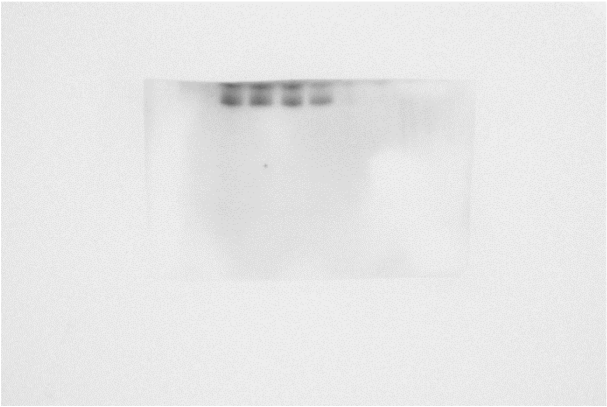

NFATc1

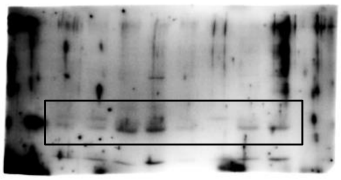

α-tubulin

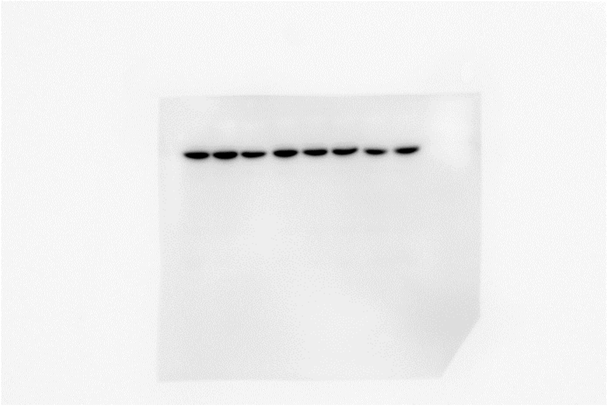

Figure S4. Presentation of original immunoblot shown in Figure 3g.
The cropped parts of immunoblot were indicated with black boxes.

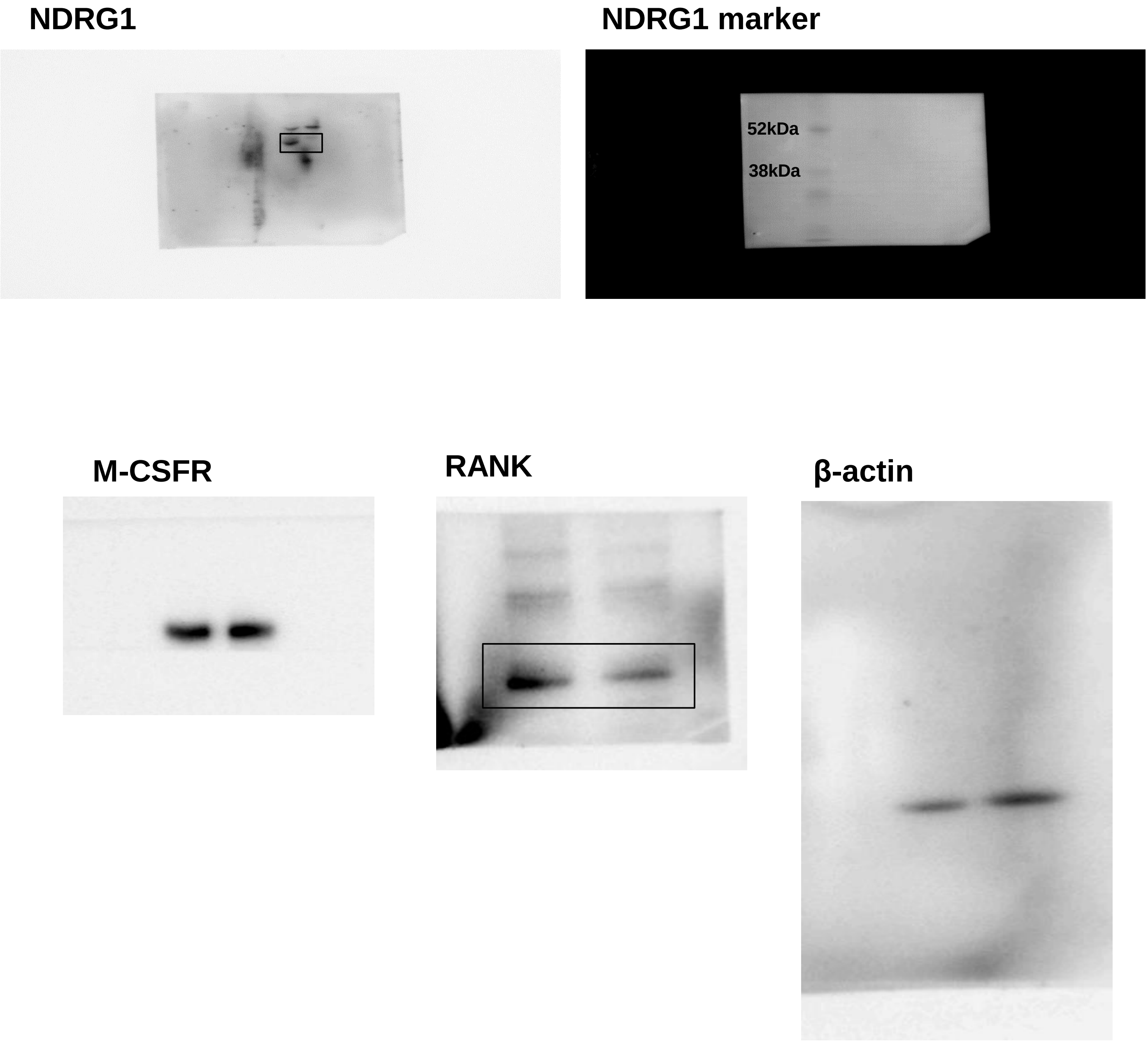

Figure S5. Presentation of original immunoblot shown in Figure 3h. The cropped parts of immunoblot were indicated with black boxes.

Supplementary methods

Cells. B16/BL6 cells and LLC/3LL cells were obtained from the RIKEN Bio Resource Center (Ibaraki, Japan). Cells were cultured in RPMI 1640 medium with 10% fetal bovine serum (FBS). To prepare B16/BL6 conditioned medium (CM), B16/BL6 cells were grown to 90% confluence, and cells were then incubated with fresh complete medium for 24 h, and the medium was collected, filtered (0.22 μm) and stored at -20°C.

Isolation of Mouse Embryonic Fibroblasts (MEFs). MEFs were prepared as previously described[41]. MEFs were cultured in Dulbecco's Modified Eagle's Medium (DMEM) (Gibco, Grand Island, NY) supplemented with 10% FBS and 4.5 g/l D-glucose.

Western Blot Analysis. Cells were rinsed with ice-cold PBS and lysed in buffer containing 50 mM of Tris-HCl, 350 mM of NaCl, 0.1% Nonidet P-40, 5 mM of ethylenediaminetetraacetic acid (EDTA), 50 mM of NaF, 1 mM phenylmethylsulfonyl fluoride, 10 μg/mL of leupeptin, and 1 mM of Na_3VO_4. Cell lysates were subjected to Western blotting as described previously[15]. The intensity of luminescence was quantified by using a charge-coupled device camera combined with an image-analysis system (LAS-4000, GE Healthcare Life Science, Little Chalfont, UK).

Tartrate-resistant acid phosphatase (TRAP) staining. At the time of euthanasia, we dissected and fixed femurs with intact periosteum in 10% buffered formalin for 48 h, decalcified them in 10% EDTA (pH 7.4) for 21 days and then embedded them in paraffin or optimal cutting temperature (OCT) compound. We processed four-micrometer-thick coronally oriented sections of bone that included the metaphysis and diaphysis for TRAP staining using a staining kit (Sigma-Aldrich). BMDMs were seeded at 5×10^4 cells with DMEM containing 10% FBS and 30 ng/mL M-CSF with or without RANKL (60 or 200 ng/mL) for 8 days. We quantified TRAP activity in the cultured pre-osteoclasts and mature osteoclasts using a TRAP staining kit (Sigma-Aldrich).

Immunohistochemical analysis and immunofluorescence. For immunofluorescence analysis of the bone sections, we incubated bone sections with primary antibodies to mouse osteocalcin (Takara, 1:200) overnight at 4°C. Subsequently, we incubated the sections with a CF488-conjugated anti-rabbit IgG at room temperature for 30 min in the dark. In each mouse in each group, we counted the numbers of positively stained cells in the whole visual field containing the distal metaphysis. Frozen sections were washed in PBS and incubated with anti-F4/80, anti-CD31 and anti-VEGF-A antibodies in an

antibody dilution solution (Dako) overnight at 4°C. Sections were then washed in PBS and incubated with CF488-conjugated anti-rat IgG or CF594-conjugated anti-rabbit IgG antibody for 30 min at room temperature. Tissue sections were immunohistochemically stained with anti-CD31 (PECAM-1) and anti-F4/80 antibodies and labeled using the peroxidase method (Histofine SABPO Kit; Nichirei, Tokyo, Japan). In all tissue samples, the mean number of infiltrating macrophages and the microvascular density (MVD) were calculated from five hotspots. Bright-field and fluorescence image were performed with KEYENCE BZ-8000. All counts were performed by three independent observers. The data were analyzed using the ImageJ program.

In vivo tumor growth experiments. 8–12 week old male mice were inoculated subcutaneously 2.5×10^5 B16/BL6 cells or 2.0×10^5 LLC/3LL cells and tumor diameters were measured with calipers in two perpendicular directions every 3 days from day 7 for 3 weeks. After mice were euthanized, tumors and lungs were harvested for immunohistochemical analysis. Tumor volumes (in mm^3) were calculated as length × $width^2 \times 0.5$.

Macrophage from Matrigel plugs. CD11b+ cells were isolated from Matrigel

plugs by magnetic sorting using CD11b MicroBeads (Miltenyi Biotec GmbH, Bergisch-Gladbach, Germany)[25]. In brief, Matrigel minced in PBS were incubated in collagenase L (Nitta Gelatin, Osaka, Japan) and DNase I (Roche Diagnostics GmbH, Mannheim, Germany) at final concentrations of 0.5% and 20 U/mL. The mixture was incubated for 1 h at 37°C under gentle agitation. The cell suspension was washed and then passed through a 70 µm mesh nylon screen. The cells were incubated with CD11b MicroBeads for 15 min at 4°C and loaded onto a MIDIMACS column (Miltenyi Biotec GmbH) according to the manufacturer's instructions. Isolated CD11b+ cells from Matrigel were used as tumor-associated macrophages (TAMs) for further experiments.

Quantitative real-time polymerase chain reaction (qRTPCR). Total RNA was isolated from cell culture using ISOGEN reagent (Nippon Gene Co. Ltd., Tokyo, Japan) according to the manufacturer's instructions, as described previously[16]. The primer pairs and probes were obtained from Applied Biosystems (Princeton, NJ). The thermal cycle conditions included maintaining the reactions at 48°C for 15 min and at 95°C for 10 min, and then alternating for 40 cycles between 95°C for 15 s and 60°C for 1 min. Results were collected and analyzed with an ABI Prism 7300 Sequence Detector System using the comparative ΔCt methods. All data were controlled for quantity of RNA input by

performing measurements on an endogenous reference gene (GAPDH).

Phagocytosis assay. BMDMs were seeded at 5×10^4 cells per well in 96 well plates and either treated with (1 ng/mL) or remained untreated for 3 h. After the incubation, non-opsonized zymosan particles (10 μL) were added, and the cells were incubated for 2 h. The quantity of engulfed zymosan particles was determined as described in the protocol of the CytoSelect 96 well phagocytosis assay from Cell Biolabs (San Diego, CA). The absorbance was measured at 405 nm in a 96 well plate reader (Beckman Coulter; Kraemer Boulevard Brea, CA).

Corneal Micropocket Assay. The corneal micropocket assay was performed essentially as described previously[42]. Briefly, 0.3 μL of Hydron pellets (Sigma-aldrich) containing human IL-1β (30 ng) was prepared and implanted in the corneas of 6–10 week old male mice treated with or without VEGF-A neutralizing antibody for ocular administration at every morning and evening. After 7 days, mice were sacrificed and their corneal vessels were photographed. Image was performed with Pixera Pro 600ES. Images of the corneas were recorded using Nikon Coolscan software with standardized illumination, contrast, and threshold settings, and were saved on disk.

Supplementary References

41. Taniguchi, K. *et al*. Suppression of Sproutys has a therapeutic effect for a mouse model of ischemia by enhancing angiogenesis. *PLoS One*. **4**, e5467 (2009).

42. Watari, K. *et al*. The antitumor effect of a novel angiogenesis inhibitor (an octahydronaphthalene derivative) targeting both VEGF receptor and NF-κB pathway. *Int. J. Cancer*. **131**, 310–321 (2012).
